# Quinine in Asthma

**Published:** 1842-05

**Authors:** B. R. Hogan

**Affiliations:** U. S. A.


*Quinine in Asthma.* By B. R. Hogan, U. S. A.—The value and importance of *quinine* in all paroxysmal and congestive diseases, have secured for it a high place in our catalogue of therapeutic agents. In the latter condition of any of the important organs, it is the safest and most efficacious agent in the materia medica. From its happy influence in relieving congestion, in our autumnal fevers, I was, *à priori,* induced to administer it in asthma. In every instance in which I have given it, or known it to be administered, the complete relief of the *dyspnœa,* and termination of the paroxysm, have succeeded in an hour. Subsequent attacks have been warded off. The paroxysm is as *tractable* to the influence of quinine as the slightest quotidian.

In the forming stage of croup, two grains of quinine, and a snuff plaster to the breast of a child two years old, parried the attack.

My friend, John A. English, M. D., of Cohaba, Ala., has used it with complete success, in a long-standing case of asthma, which had resisted the usual remedies. Four grains of quinine "operated like a charm," and relieved the patient in thirty minutes.

I communicate to you this important effect of quinine, in order that the profession may scrutinize the high claim I set up for it, and, by further experience, direct in what cases it may be most beneficially administered in one of the most distressing and intractable diseases "which flesh is heir to." From two to eight grains of the sulph. quinine, at a dose, to be repeated in an hour if not relieved.—*Am. Med. Intell.*

